# Senescent endothelial cells: key commanders of the cellular communication network within atherosclerotic plaques

**DOI:** 10.3389/fimmu.2026.1712974

**Published:** 2026-02-04

**Authors:** Fujia Xie, Cheng Xi, Guoqing Bao, Bin Yang, Xiaoyu Zheng, Bowen Fu, Zhibin Zheng

**Affiliations:** 1 General Surgery, First Affiliated Hospital of Kunming Medical University, Kunming, Yunnan, China; 2 Day Surgery Center, First Affiliated Hospital of Kunming Medical University, Kunming, Yunnan, China

**Keywords:** atherosclerosis, endothelial senescence, retinol-binding protein, senescence-associated secretory phenotype, therapeutic targeting

## Abstract

Endothelial cell senescence, once considered a passive manifestation of vascular aging, is now recognized as an active driver of atherosclerosis. Senescent endothelial cells (sECs) exhibit distinct morphological and molecular hallmarks, including irreversible growth arrest, altered chromatin structure, and secretion of a pro-inflammatory senescence-associated secretory phenotype (SASP). Through SASP factors, extracellular vesicles, and paracrine signaling, sECs orchestrate a pathological communication network that recruits immune cells, reprograms vascular smooth muscle cells, and compromises endothelial integrity, collectively promoting plaque growth and instability. Central signaling pathways such as the p53/p21 and p16/Rb axes establish the senescent state, while mTOR, NF-κB, and cGAS-STING pathways sustain SASP production. We propose the retinol-binding protein 4 (RBP4) axis as a compelling theoretical framework linking metabolic dysfunction to endothelial senescence. While the TLR4-mediated inflammatory pathway is established, we posit a convergent STRA6-mediated axis that may integrate systemic metabolic stress with local vascular inflammation. Recognizing sECs as “commanders” of the atherosclerotic microenvironment highlights their potential as therapeutic targets. Strategies including senolytics, senomorphics, and upstream pathway inhibition offer promising avenues for attenuating vascular aging. Crucially, our analysis emphasizes the necessity of sex-specific therapeutic approaches, distinguishing between inflamm-aging driven pathologies in men and mechanisms centered on metabolic resilience in women.

## 1 Section 1. the commander’s profile: hallmarks of endothelial cell senescence

This review offers three distinct contributions to the field. First, we conceptualize the senescent endothelial cell not as a passive bystander but as an active ‘Commander’ and ‘Tissue Engineer’ of the plaque. Second, we integrate the novel RBP4-STRA6 signaling axis as a critical bridge linking systemic metabolic dysfunction (e.g., obesity) directly to local vascular senescence. This framework offers a biological basis for understanding how metabolic stress translates into cell cycle arrest. Finally, incorporating recent single-cell clinical data (1), we critically analyze the sexual dimorphism of endothelial senescence, highlighting why ‘inflamm-aging’ is a predominant driver in men but not women. This synthesis provides a roadmap for developing sex-specific precision therapies, such as targeted Antibody-Drug Conjugates (ADCs) (2), moving beyond generic anti-inflammatory strategies.

### 1.1 Morphological and functional transformation: from quiescent guardian to dysfunctional instigator

Senescent endothelial cells undergo dramatic physical and functional transformations that compromise vascular integrity. Morphologically, they become flattened, enlarged, and often multinucleated, losing their typical ‘cobblestone’ architecture (3). This structural deterioration directly leads to functional barrier failure: the loss of tight junctions creates a ‘leaky’ endothelium that facilitates the subendothelial infiltration of lipids (e.g., LDL) and immune cells (4, 5).This combination of enhanced persistence and barrier dysfunction allows sECs to establish long-term, pathological command posts within the vessel wall.”

### 1.2 Molecular fingerprint: core biomarkers

Senescent endothelial cells possess a range of detectable molecular markers. A key biochemical hallmark is the enhanced activity of senescence-associated β-galactosidase (SA-β-gal) at a non-optimal pH of 6.0, which stems from a significant expansion of the lysosomal compartment (6). The core molecules driving the senescent cell cycle arrest are the cyclin-dependent kinase inhibitors (CDKIs), particularly p16INK4a and p21Waf1/Cip1. Their upregulated expression is a defining molecular feature of senescence (5). Other markers include the formation of telomere-associated foci, altered chromatin structure (senescence-associated heterochromatin foci, SAHF), and the loss of nuclear Lamin B1 (**Table 1**) (6).

**Table 1:** Hallmarks and biomarkers of senescent endothelial cells.

Category	Marker/feature	Description of change	Key references
Morphology	Cellular hypertrophy, flattening	Loss of typical “cobblestone” morphology, increased size	(5)
Cell Cycle	SA-β-gal activity	Increased activity at pH 6.0	(6)
	p16INK4a expression	Upregulated, leading to dephosphorylation of Rb protein	(6)
	p21Waf1/Cip1 expression	Upregulated in response to DNA damage	(6)
	Proliferative arrest	Irreversible cell cycle arrest	(5)
Secretome	Senescence-Associated Secretory Phenotype (SASP)	Secretion of pro-inflammatory cytokines, chemokines, proteases	(9)
Function	Barrier permeability	Loss of tight junctions, increased permeability	(4)
	NO bioavailability	Decreased eNOS expression, impaired vasodilation	(5)
	Adhesion molecule expression	Upregulation of VCAM-1, ICAM-1	(17)
	Response to blood flow	Loss of ability to align with laminar shear stress	(5)

### 1.3 Origins of senescence: a response to pro-atherogenic stress

Endothelial cell senescence is not a random event but a direct response to various stressors prevalent in the cardiovascular system (5).

#### 1.3.1 Hemodynamic stress

Disturbed, low, and oscillatory shear stress, present at arterial bifurcations and curvatures, is a primary factor promoting endothelial senescence, which explains the focal nature of atherosclerotic plaques (5). These mechanical forces induce senescence by activating signaling pathways such as p53-p21 (7).

#### 1.3.2 Oxidative and genotoxic stress

Factors such as reactive oxygen species (ROS), components of cigarette smoke (e.g., benzo[a]pyrene), and unrepaired DNA damage trigger a potent DNA damage response (DDR), ultimately leading to senescence (7). The p53 and cGAS-STING pathways are key mediators of this response (7).

#### 1.3.3 Metabolic stress

Pathological states like hyperglycemia, hyperlipidemia (e.g., oxidized LDL), and elevated levels of certain adipokines can directly induce or accelerate endothelial cell senescence (8).

#### 1.3.4 Telomere shortening

**Figure 1:**
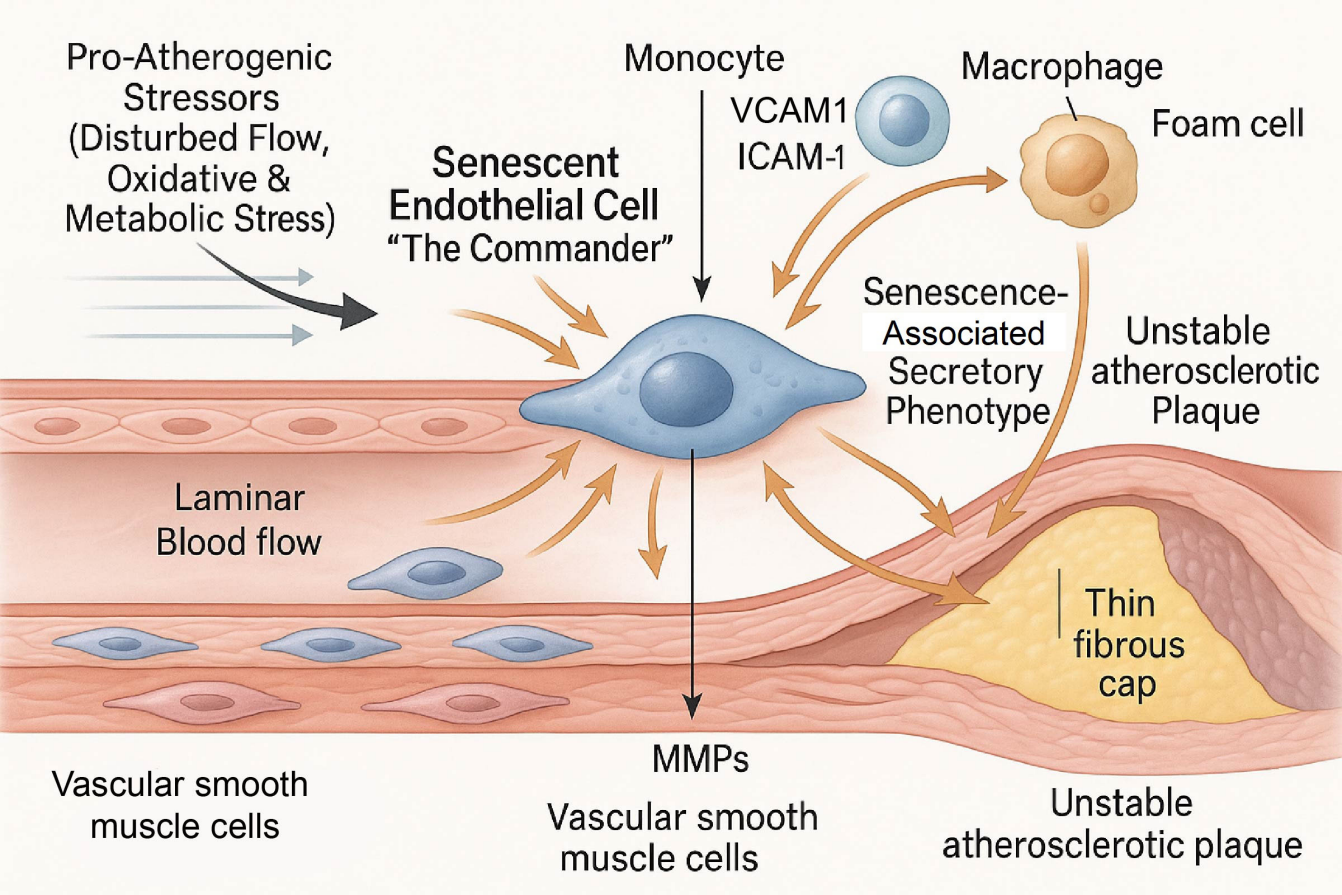
The senescent endothelial cell (sEC) as a central commander in the pathogenesis of atherosclerosis. This schematic diagram illustrates the process by which pro-atherogenic stressors, such as disturbed blood flow and metabolic stress, induce endothelial cell senescence. The resulting sEC transitions into a pathological “commander,” actively orchestrating plaque development. The sEC secretes a complex mixture of pro-inflammatory factors known as the senescence-associated secretory phenotype (SASP). Concurrently, the sEC upregulates surface adhesion molecules (VCAM-1, ICAM-1). Together, these signals recruit circulating monocytes, which then transmigrate into the subendothelial space, differentiate into macrophages, and become lipid-laden foam cells. Key components of the SASP, such as matrix metalloproteinases (MMPs), degrade the extracellular matrix of the fibrous cap, thereby contributing to plaque instability and the formation of a vulnerable plaque phenotype. sEC, senescent endothelial cell; SASP, senescence-associated secretory phenotype; VCAM-1, vascular cell adhesion molecule 1; ICAM-1, intercellular adhesion molecule 1; MMPs, matrix metalloproteinases.

Although endothelial cells are typically quiescent, in areas of high turnover due to injury or disturbed flow, cells undergo replicative senescence. This process is driven by the progressive shortening of telomeres, which itself activates the DNA damage response (**Figure 1**) (6).

## 2 Section 2. the commander’s arsenal: mechanisms of intercellular communication

The communication strategy of a senescent endothelial cell is not singular but a multi-layered, complex system designed for robust and persistent control. It combines wide-area broadcasting (SASP), targeted delivery (EVs), and signal amplification (paracrine senescence) to ensure its pro-atherogenic directives are not only received but also sustained and amplified, overwhelming the tissue’s homeostatic and repair mechanisms.

### 2.1 Senescence-associated secretory phenotype: the primary communication channel

The SASP is the sEC’s primary weapon, transforming a single, arrested cell into a potent signaling hub that profoundly alters the tissue microenvironment (9). It is a complex, heterogeneous secretome whose specific composition depends on the cell type and the senescence-inducing stimulus (10).

#### 2.1.1 Composition: a pro-inflammatory cocktail

The SASP is rich in pro-inflammatory cytokines (e.g., IL-6, IL-1α, IL-1β, TNF-α), chemokines (e.g., IL-8, MCP-1/CCL2), and growth factors.2 These molecules act as “broadcast signals,” creating a state of chronic, low-grade inflammation (“inflammaging”) within the vessel wall (11).

#### 2.1.2 The proteolytic arm: matrix metalloproteinases

A key component of the SASP is a suite of proteases, particularly matrix metalloproteinases (e.g., collagenases, stromelysins). These enzymes degrade the extracellular matrix (ECM), directly contributing to the structural weakening of the plaque’s fibrous cap (**Table 2**) (6).

**Table 2:** Key components of the endothelial SASP and their functions in atherosclerosis.

SASP component	Category	Pro-atherogenic function	Key references
IL-6, TNF-α	Cytokines	Promote systemic and local inflammation, induce paracrine senescence	(14)
MCP-1 (CCL2)	Chemokines	Recruits monocytes from the bloodstream to the subendothelial layer	(17)
VCAM-1, ICAM-1	Adhesion Molecules	Mediate adhesion of circulating leukocytes (primarily monocytes)	(17)
MMPs (e.g., MMP-9, MMP-13)	Proteases	Degrade the extracellular matrix (e.g., collagen) of the fibrous cap, leading to plaque instability	(57)
IL-1α, IL-1β	Cytokines	Potent pro-inflammatory signals that can amplify SASP production	(17)
Growth Factors (e.g., VEGF)	Growth Factors	May influence angiogenesis and cell proliferation	(21)

### 2.2 Extracellular vesicles and exosomes: packaged directives for the microenvironment

In addition to soluble factors, sECs release membrane-bound extracellular vesicles, including microvesicles and smaller exosomes (30–150 nm in diameter) (12). These act as “packaged directives,” carrying a concentrated cargo of proteins, lipids, and nucleic acids (mRNA, miRNA) for targeted delivery to recipient cells (13). EVs from senescent or damaged endothelial cells can mediate intercellular communication, promoting inflammation, thrombosis, and vascular calcification (12). They are capable of transferring their cargo to neighboring cells, such as vascular smooth muscle cells (VSMCs) or macrophages, thereby altering their function in a targeted manner (13).

### 2.3 Paracrine senescence: spreading the senescent phenotype to amplify command

**Figure 2:**
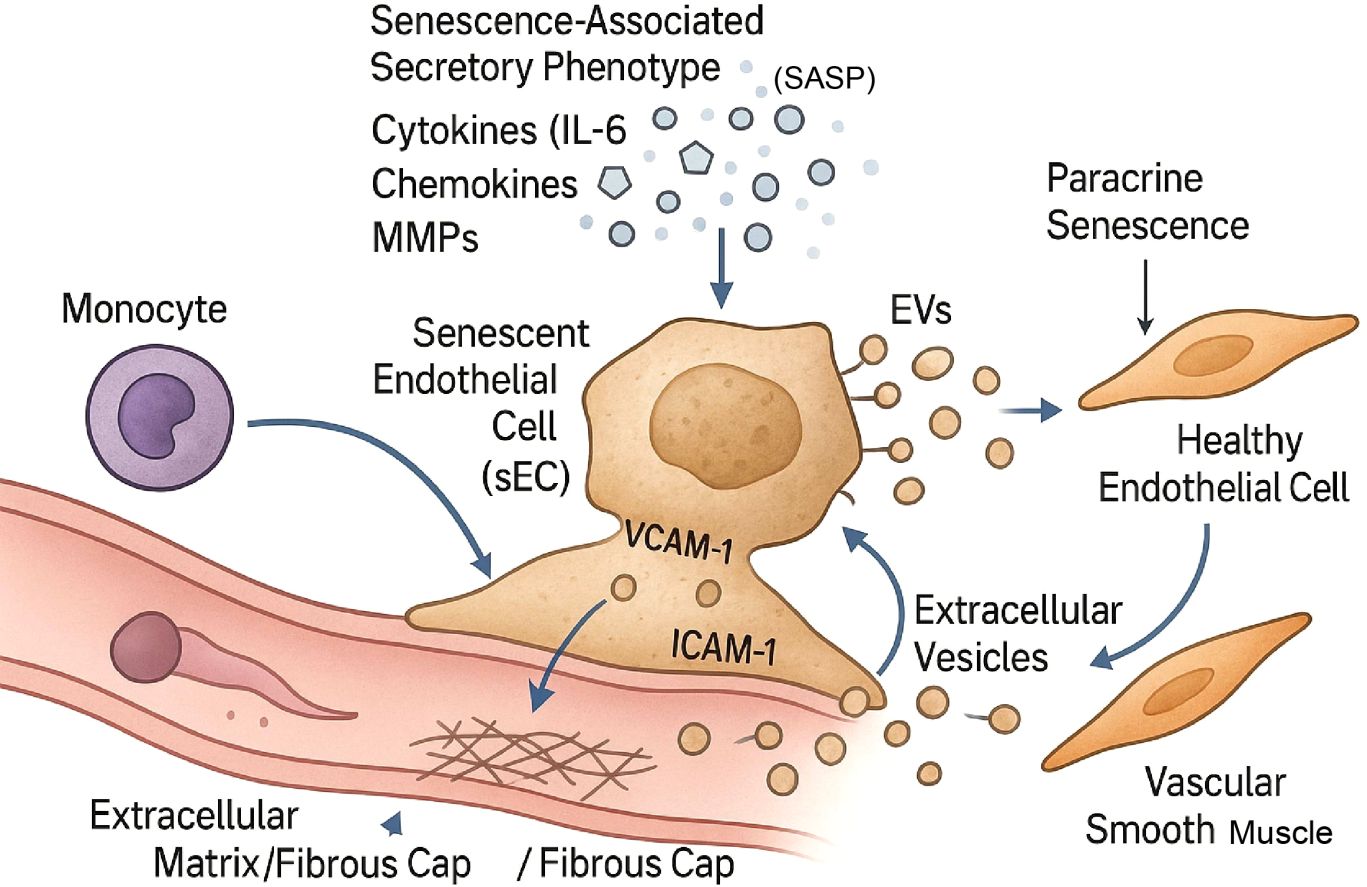
Mechanisms of intercellular communication employed by the senescent endothelial cell (sEC). The sEC utilizes multiple communication channels to modulate its microenvironment. The primary mechanism is the secretion of the senescence-associated secretory phenotype (SASP), a heterogeneous mixture of soluble factors including pro-inflammatory cytokines (e.g., IL-6), chemokines, and proteases (e.g., MMPs). In parallel, sECs release extracellular vesicles (EVs) containing bioactive cargo that can be delivered to target cells, such as vascular smooth muscle cells (VSMCs), to alter their function. The sEC surface expresses adhesion molecules (VCAM-1, ICAM-1) that mediate the capture of circulating monocytes. Furthermore, factors within the SASP can induce paracrine senescence in adjacent healthy endothelial cells, thereby amplifying the pro-atherogenic signal field. sEC, senescent endothelial cell; SASP, senescence-associated secretory phenotype; IL-6, interleukin-6; MMPs, matrix metalloproteinases; EVs, extracellular vesicles; VCAM-1, vascular cell adhesion molecule 1; ICAM-1, intercellular adhesion molecule 1.

A highly destructive capability of the SASP is the induction of “paracrine senescence” or “bystander senescence.” Inflammatory factors released by sECs can trigger senescence in adjacent healthy cells, including other endothelial cells and vascular smooth muscle cells (14). This creates a vicious, self-amplifying cycle. One sEC can “recruit” its neighbors, turning them into new secretory commanders, thereby spreading and reinforcing the pro-atherogenic signaling field throughout the lesion (15). Key mediators of this process include transforming growth factor-beta (TGF-β) and reactive oxygen species (ROS), which are both components of and can be induced by the SASP (**Figure 2**) (16). However, this complex secretory network is not merely a byproduct of cellular decay; it serves as a sophisticated tool for active environmental remodeling. This capability directly enables the sEC to transition from a localized damaged cell to a central regulatory hub that actively modulates the behavior of other plaque components, as detailed in the following section.”

## 3 Section 3. the commander’s directives: impact on key cells within the atherosclerotic plaque

The role of the sEC extends beyond that of a mere inflammation promoter. It acts as a malignant tissue engineer, actively remodeling the vessel wall to favor plaque growth and instability. It commands the behavior of monocytes (recruitment, infiltration) and precisely modulates the behavior of VSMCs, instructing them to cease collagen production and instead produce MMPs (17). This series of highly specific directives leads to a predictable physical outcome: a lipid-rich inflammatory core (from foam cells) covered by a thin, unstable fibrous cap (from reprogrammed VSMCs). Functionally, the sEC acts akin to a maladaptive “engineer.” Representing a breakdown of homeostatic control, it provides a pathological blueprint that misdirects other cells (“workers”) to construct a flawed structure—the unstable atherosclerotic plaque.

### 3.1 Commanding immune cells: orchestrating monocyte recruitment and chronic inflammation

Senescent endothelial cells upregulate cell adhesion molecules like VCAM-1 and ICAM-1 on their surface (17). These molecules act as “docking sites” for circulating monocytes. Simultaneously, the SASP, rich in chemokines like MCP-1 (CCL2), provides a chemical “beacon” that actively recruits these monocytes from the bloodstream into the subendothelial space (17). Once in the intima, these monocytes differentiate into macrophages, which become foam cells after engulfing oxidized lipids—the hallmark of early atherosclerotic fatty streaks. By orchestrating this entire process, the sEC acts as the primary gatekeeper driving chronic inflammation. However, the sEC’s influence extends beyond the innate immune system to the adaptive branch. Insights from oncology offer a compelling template for understanding this spatial complexity. Recent spatial transcriptomic analyses in tumor microenvironments have characterized “immune niches” driven by interactions between SPP1+ macrophages and exhausted T-cells (18–20). We postulate that sECs may function similarly to stromal organizers in these niches. By secreting CXCL10/11 while simultaneously upregulating PD-L1, sECs have the potential to not only recruit T-cells but also induce their exhaustion or Treg dysfunction, mirroring the immunosuppressive landscape observed in tumors (17, 18). This “tumor-like” immune plasticity represents a critical, yet under-explored, dimension of plaque instability. Additionally, sEC-derived chemokines (e.g., CXCL8) actively recruit neutrophils, triggering the release of Neutrophil Extracellular Traps (NETs). Crucially, the bioactive components of NETs (e.g., histones, cell-free DNA) act as secondary senescence-inducing signals, likely re-activating the cGAS-STING pathway in endothelial cells. This establishes a self-sustaining vicious cycle of inflammation and damage that persists even after the initial stressor is removed. Furthermore, drawing parallels from tumor microenvironment studies, recent evidence suggests that sECs may also orchestrate diverse immune niches, potentially influencing the plasticity of immune cells beyond simple recruitment (18, 20). By orchestrating this entire process—from initial recruitment to the shaping of complex immune landscapes—the sEC acts as the primary gatekeeper driving the chronic inflammation that fuels plaque growth.

### 3.2 Manipulating vascular smooth muscle cells: engineering plaque instability

In a healthy vessel, VSMCs provide structural integrity. However, sECs reprogram them for a destructive role. SASP factors can induce VSMC senescence (21). Senescent VSMCs undergo a phenotypic switch: they reduce their secretion of ECM proteins (like collagen) that are vital for the strength of the protective fibrous cap of the plaque (22). Concurrently, they increase their secretion of matrix-degrading enzymes, particularly MMPs, which actively digest the fibrous cap, making it thinner, more fragile, and prone to rupture—the key event that triggers acute thrombotic events like myocardial infarction and stroke (23).

### 3.3 Compromising endothelial integrity: autocrine reinforcement and barrier dysfunction

The SASP has autocrine effects, meaning the factors secreted by an sEC can act on itself, thereby reinforcing the senescent state and resistance to apoptosis (24). By inducing paracrine senescence in neighboring endothelial cells, the initial sEC spreads dysfunction along the endothelial monolayer, leading to widespread barrier failure, impaired vasodilation (due to reduced eNOS expression in sECs), and the formation of a pro-thrombotic surface (6).

## 4 Section 4. the commander’s command center: key signaling pathways driving senescent endothelial cell function

### 4.1 Foundational pathways of senescence: the p53/p21 and p16INK4a/Rb axes

These two tumor suppressor pathways are the core machinery that executes the cell cycle arrest defining senescence (25).

#### 4.1.1 The p53/p21 pathway: the first responder

In response to acute stressors like DNA damage or disturbed flow, the p53 protein is stabilized and activated (26). Activated p53 transcriptionally upregulates the CDKI p21. p21 then inhibits cyclin-dependent kinases (primarily CDK2), leading to an initial, sometimes reversible, cell cycle arrest (27). In endothelial cells, this pathway is a key mediator of senescence induced by disturbed flow (28). p21 also plays a crucial role in maintaining the viability of senescent cells, preventing their apoptosis (29).

#### 4.1.2 The p16INK4a/Rb pathway: the enforcer of irreversible arrest

Under conditions of sustained or severe stress, the p16INK4a (p16) pathway is activated. p16 specifically inhibits CDK4/6, preventing the phosphorylation of the retinoblastoma (Rb) protein. Hypophosphorylated Rb remains active and sequesters E2F transcription factors, thereby robustly blocking cell entry into the S phase (30). Activation of p16 is generally considered the key step that locks a cell into an irreversible senescent state (31). Furthermore, p16 and p21 act cooperatively to establish and maintain this arrest (32).However, the therapeutic manipulation of this pathway requires caution. While p16 exerts its effects by inhibiting CDK4/6, recent *in vivo* evidence suggests that pharmacological inhibition of CDK4/6 (e.g., using Palbociclib) does not alleviate vascular aging but paradoxically exacerbates endothelial senescence and atherosclerosis (33). Hu et al. demonstrated that suppressing CDK4/6 downstream of CDKN2A led to increased ROS production and cell cycle arrest characteristic of a senescent phenotype, rather than protecting the vessel (33). This reveals a fundamental logical distinction: cell cycle arrest is a symptom of the damage response, not the cause. Forcing a stressed cell to arrest via CDK4/6 inhibition without resolving the upstream stressor (e.g., DNA damage) does not preserve function; instead, it locks the cell into a dysfunctional, senescent state. Thus, therapy must target the upstream triggers rather than the downstream brake mechanism. Activation of p16 is generally considered the key step that locks a cell into an irreversible senescent state and is a robust biomarker of senescence. p16 and p21 act cooperatively to establish and maintain the senescent state.

### 4.2 Fueling the arsenal: upstream regulation of the SASP

The production of the SASP is not a passive byproduct of arrest but an active process regulated by specific signaling pathways.

#### 4.2.1 The mTOR pathway: the master metabolic regulator of SASP synthesis

The mechanistic target of rapamycin (mTOR) pathway is a central hub that integrates nutrient and growth signals. In senescent cells, mTORC1 is persistently active and is critical for the synthesis and secretion of many SASP components, particularly by controlling the translation of key mRNAs like that for IL-1α (34). Inhibition of mTOR with rapamycin can suppress the SASP without reversing the growth arrest, making it a key “senomorphic” target (35).

#### 4.2.2 The NF-κB and cGAS-STING pathways: translating damage into inflammation

**Figure 3:**
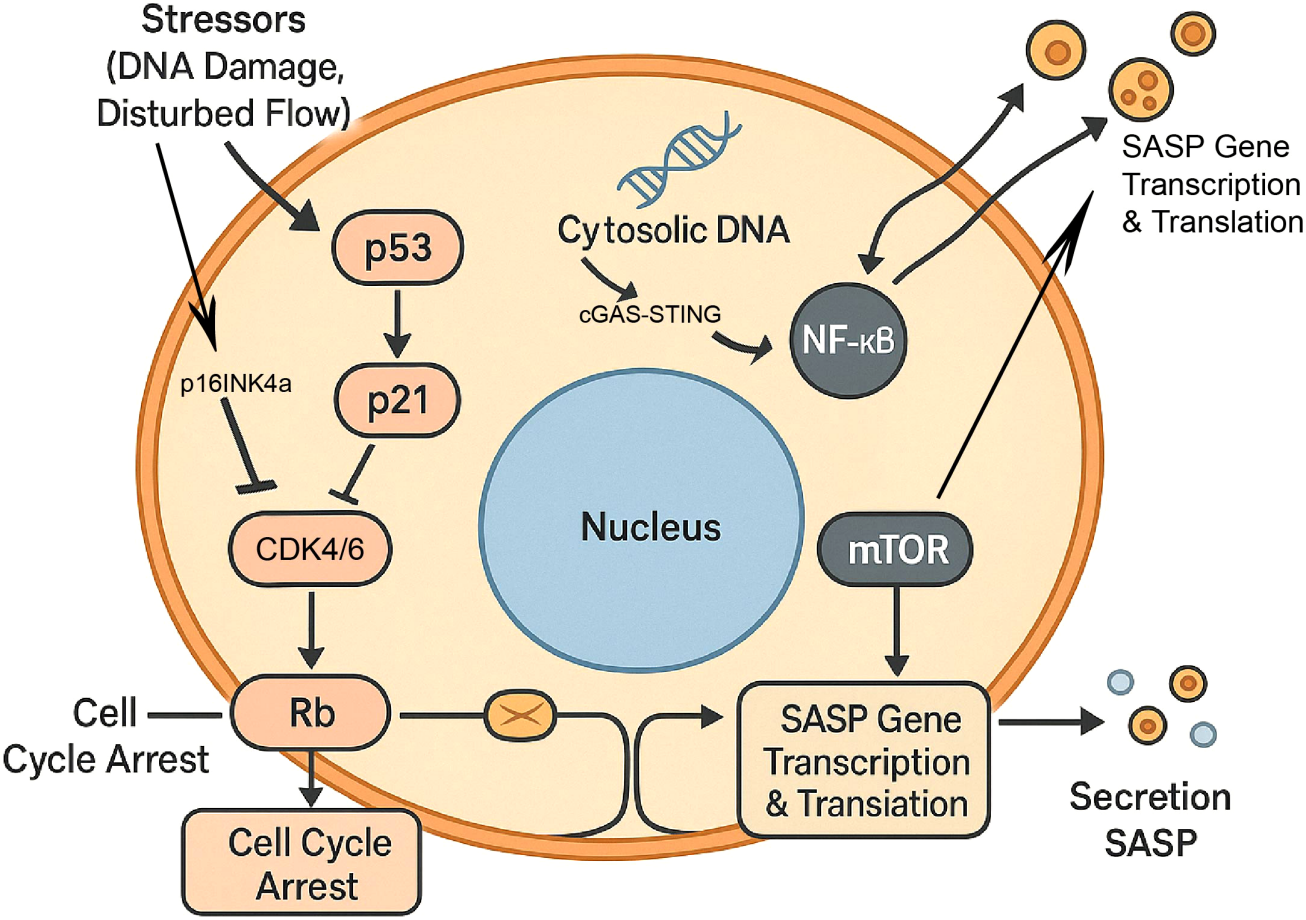
Core intracellular signaling pathways driving the senescent endothelial cell (sEC) phenotype. The sEC phenotype is established and maintained by two distinct sets of parallel signaling pathways. (1) Cell Cycle Arrest: Arrest is enforced by two cooperative tumor suppressor pathways. The p53-p21 axis is activated by stressors, where p53 upregulates p21. In parallel, the p16INK4a pathway is activated by sustained stress. Both p21 and p16INK4a inhibit cyclin-dependent kinases (CDKs), such as CDK4/6, leading to the activation of the retinoblastoma (Rb) protein and subsequent cell cycle arrest. (2) SASP Regulation: The pro-inflammatory SASP is driven by two parallel regulatory arms. The cGAS-STING pathway senses cytosolic DNA and activates the transcription factor NF-κB, a master regulator of SASP gene expression. Concurrently, the mTOR pathway, a central metabolic regulator, is persistently active and promotes the synthesis and translation of SASP components. sEC, senescent endothelial cell; CDK, cyclin-dependent kinase; Rb, retinoblastoma protein; SASP, senescence-associated secretory phenotype; cGAS, cyclic GMP-AMP synthase; STING, stimulator of interferon genes; NF-κB, nuclear factor kappa-light-chain-enhancer of activated B cells; mTOR, mechanistic target of rapamycin.

The transcription factor NF-κB is a master regulator of inflammation and a key driver of SASP gene expression (36). It is activated by a multitude of senescence-inducing stimuli. A key upstream activator is the cGAS-STING pathway, which senses cytosolic DNA (e.g., from damaged mitochondria or micronuclei) and triggers a potent inflammatory response, including the SASP (**Figure 3**) (37).

### 4.3 Special focus: the RBP4 axis—a novel command and control pathway

This section details a critical emerging pathway that directly links metabolic dysfunction, a primary risk factor for atherosclerosis, to endothelial senescence and inflammation. While clinical epidemiology identifies risk factors (e.g., obesity, diabetes), molecular biology uncovers mechanisms (senescence, inflammation). RBP4 is a molecule that bridges this gap. It is not merely correlated with risk factors; it is a mechanistic mediator produced by the risk-factor state (e.g., hypertrophied adipose tissue) that directly executes a pathological mechanism (endothelial inflammation and senescence) at the vessel wall. This unique position makes the RBP4 axis a highly attractive target, as its inhibition could simultaneously address the consequences of multiple major cardiovascular risk factors.

#### 4.3.1 RBP4: the adipokine linking metabolic disease to vascular inflammation

Retinol-binding protein 4 (RBP4) is an adipokine whose circulating levels are elevated in obesity, type 2 diabetes, and metabolic syndrome (38). High levels of RBP4 are associated with CVD, atherosclerosis, and endothelial dysfunction, making it a key molecular bridge connecting these conditions (39).

#### 4.3.2 Duality of RBP4 receptor signaling: STRA6 vs. TLR4

The action of RBP4 is complicated by its interaction with at least two different receptor systems.

STRA6 (Stimulated by Retinoic Acid 6): The classic receptor for holo-RBP4 (all-trans-retinol-RBP4), which mediates retinol transport into the cell (40).

TLR4 (Toll-like Receptor 4): A pattern recognition receptor of the innate immune system. Crucially, studies in endothelial cells have shown that RBP4 can induce inflammation via TLR4, a process that is independent of retinol and STRA6 (41).

#### 4.3.3 The STRA6-JAK-STAT cascade: a retinol-dependent pathway with pro-senescent crosstalk

The binding of holo-RBP4 to STRA6 not only transports retinol but also activates a signaling cascade similar to that of cytokine receptors. The activation of Janus kinase 2 (JAK2) and subsequent phosphorylation of STAT proteins (STAT3/5) is a well-characterized downstream effect of STRA6 signaling (42). Although direct evidence of this specific cascade driving senescence within endothelial cells is still emerging, studies in adipocytes and cancer models have solidly established a crosstalk between STAT signaling and the p53 pathway (43). Based on these parallels, we propose a convergent model for the atherogenic endothelium: RBP4-induced STAT activation could theoretically upregulate SOCS proteins to stabilize p53, thereby reinforcing cell cycle arrest (44). While direct confirmation of this specific cascade in vascular endothelium warrants further investigation, it offers a biologically plausible framework linking metabolic signaling to cell cycle control. Validating this specific axis is crucial not only for mechanism but for precision medicine. Given that female endothelial cells exhibit superior transcriptomic signatures of metabolic regulation (1), it is plausible that sex-specific resilience against this RBP4-STRA6 metabolic stress axis contributes to the sexual dimorphism observed in plaque progression. This highlights a potential divergence point where male endothelium may be more susceptible to metabolic-driven senescence. Furthermore, this metabolic stress likely converges with mitochondrial dysfunction and oxidative stress loops recently described in vascular aging (45), creating a self-sustaining cycle of senescence.”

#### 4.3.4 The TLR4-NF-κB/MAPK cascade: a STRA6-independent inflammatory pathway in endothelial cells

In endothelial cells, which may express low levels of STRA6, RBP4 acts as a damage-associated molecular pattern (DAMP) (41). It binds to TLR4, activating downstream inflammatory pathways including NF-κB and MAP kinases (JNK, p38) (46). This directly stimulates the expression of pro-inflammatory SASP components (e.g., VCAM-1, ICAM-1, and IL-6), promoting endothelial inflammation and leukocyte adhesion (47). This pathway is potent and independent of RBP4’s retinol-carrying status (48).

#### 4.3.5 Synthesis: how RBP4 drives endothelial senescence and atherosclerosis via convergent pathways

**Figure 4:**
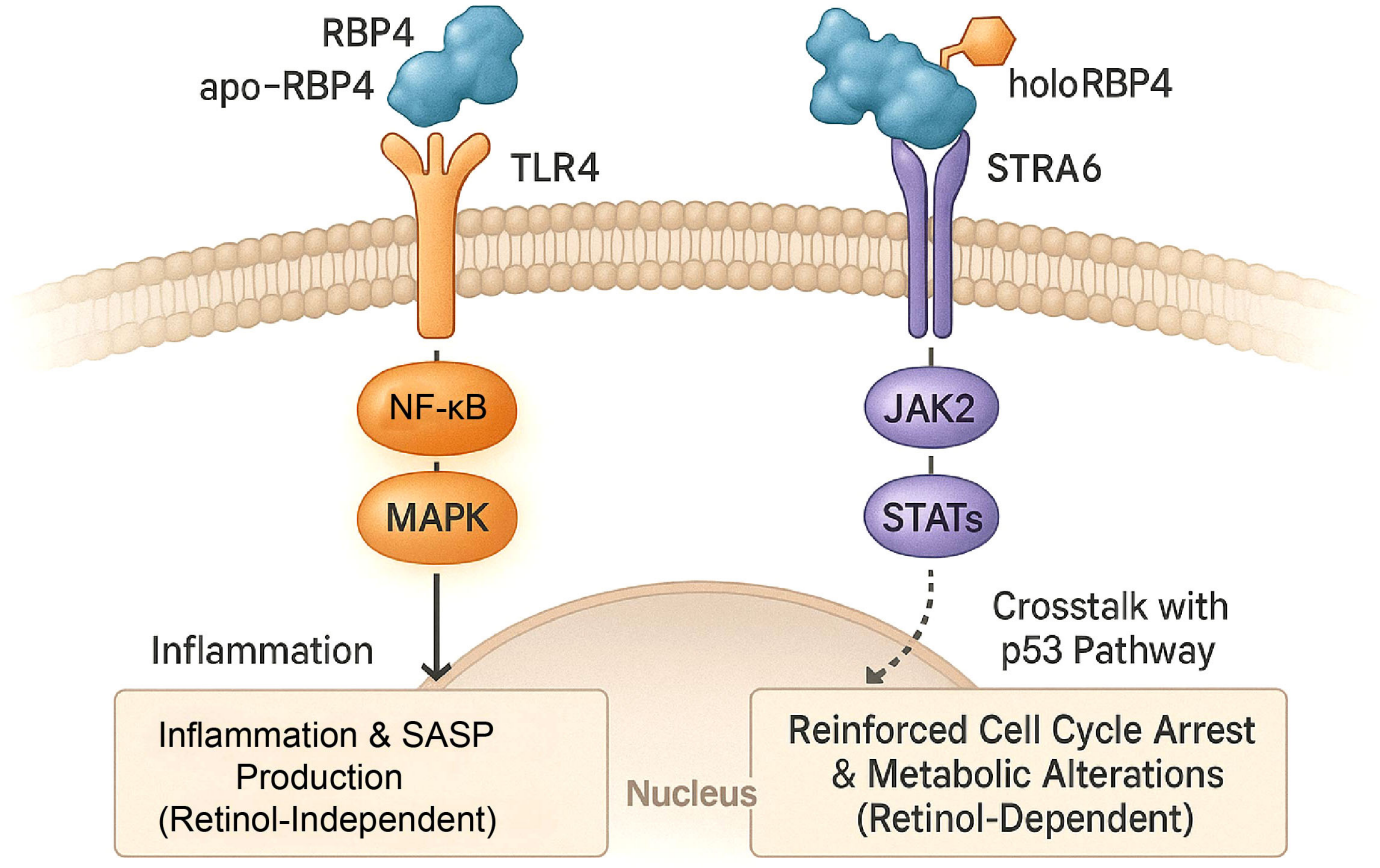
The dual receptor signaling pathways of retinol-binding protein 4 (RBP4) in endothelial cells. The adipokine RBP4 integrates metabolic and inflammatory signals through two distinct receptor pathways. (1) Retinol-Independent Pathway: Both apo-RBP4 and holo-RBP4 can bind to Toll-like receptor 4 (TLR4). This engagement activates downstream inflammatory cascades involving NF-κB and MAP kinases (MAPK), leading to inflammation and SASP production. (2) Retinol-Dependent Pathway: Only holo-RBP4 (retinol-bound RBP4) binds to its classic receptor, STRA6. This interaction activates the JAK2-STATs signaling cascade. Based on evidence from adipocytes and cancer models, we propose a hypothetical crosstalk mechanism where STAT activation may upregulate SOCS proteins to stabilize p53. While this specific interaction remains to be validated in endothelial cells, it represents a plausible link reinforcing the senescent state. The convergence of these two pathways makes RBP4 a potent driver of the sEC phenotype. RBP4, retinol-binding protein 4; TLR4, Toll-like receptor 4; NF-κB, nuclear factor kappa-light-chain-enhancer of activated B cells; MAPK, mitogen-activated protein kinase; SASP, senescence-associated secretory phenotype; STRA6, stimulated by retinoic acid 6; JAK2, Janus kinase 2; STATs, Signal Transducer and Activator of Transcription proteins.

RBP4 uniquely drives endothelial senescence through a two-pronged attack. The TLR4-mediated pathway provides a rapid, direct route to inflammation, a key component of the sEC phenotype. The STRA6-JAK-STAT pathway, driven by metabolic dysregulation, provides a separate input that can reinforce the core cell cycle arrest machinery through crosstalk with p53. This convergence creates a “two-hit” pathological mechanism: hemodynamic stress at arterial bifurcations likely primes the endothelium (e.g., via receptor upregulation or glycocalyx disruption), rendering these focal areas uniquely hypersensitive to circulating systemic RBP4. This synergy explains why systemic metabolic dysfunction translates into localized vascular senescence, solidifying the “commander” role of the sEC specifically in the athero-prone environment (**Figure 4**, **Table 3**).

**Table 3:** Comparative analysis of RBP4 signaling pathways in vascular cells.

Feature	Pathway 1: TLR4-mediated signaling	Pathway 2: STRA6-mediated signaling
Receptor	Toll-like receptor 4 (TLR4)	Stimulated by retinoic acid 6 (STRA6)
Primary Ligand	Apo-RBP4 and Holo-RBP4	Holo-RBP4 only (retinol-bound)
Key Downstream Mediators	NF-κB, MAP kinases (JNK, p38)	Janus kinase 2 (JAK2), STAT proteins (STAT3, STAT5)
Primary Cellular Outcome	Inflammation, SASP production, adhesion molecule upregulation	Insulin resistance, metabolic alterations, crosstalk with p53 pathway
Retinol Dependency	Independent	Dependent
Key Cell Types	Endothelial cells, macrophages	Adipocytes, pancreatic β-cells, other parenchymal tissues
Role in Atherosclerosis	Directly induces endothelial inflammation and dysfunction	Indirectly contributes to a pro-senescent environment via metabolic dysregulation and hypothesized STAT-p53 crosstalk (mechanism extrapolated from non-endothelial models)
Key References	(58)	(59)

## 5 Section 5. dethroning the commander: therapeutic strategies targeting senescent endothelial cells

The diverse therapeutic strategies targeting senescent cells present a strategic choice that may require individualized and context-dependent solutions. Senescence has a dual role: it can be beneficial (acute, for wound healing/tumor suppression) or detrimental (chronic, in age-related diseases) (49). Therefore, the choice to “kill” (senolytics), “disarm” (senomorphics), or “prevent” (upstream inhibition) is not a one-size-fits-all decision. For patients with advanced, unstable plaques, short-term, potent senolytics may be most effective. For those with early risk factors, long-term senomorphic or upstream inhibitor therapy may be more appropriate. Sex-specific findings further underscore that the choice of strategy must be tailored to the individual’s specific pathology (50).

### 5.1 Senolytics: a targeted clearance strategy

Senolytics are a class of drugs that can selectively induce apoptosis in senescent cells, thereby clearing them from tissues (51). An example is the combination of dasatinib and quercetin (D+Q). Preclinical studies in mouse models of atherosclerosis have shown that clearing senescent cells with senolytics can reduce plaque formation, decrease inflammation, and even improve plaque stability by preventing fibrous cap thinning (52). This approach directly “dethrones” the commander. However, a critical caveat remains: sECs, despite being dysfunctional, physically cover the vessel wall. Their rapid apoptotic clearance by senolytics could theoretically expose the thrombogenic subendothelium if not matched by the regenerative capacity of adjacent healthy cells. Therefore, the clinical application of senolytics must consider the “replacement kinetics” to avoid transient plaque destabilization. Emerging clinical data suggest potential benefits but also reveal significant complexities, particularly regarding biological sex. A recent pivotal study by Mury et al. utilizing single-nuclei RNA sequencing on human internal thoracic arteries revealed a striking sexual dimorphism: endothelial cells from male patients exhibited a distinct “inflamm-aging” signature characterized by shortened telomeres, upregulated CDKN1A (p21), and robust SASP secretion (1). In contrast, female endothelial cells, even in post-menopausal patients, maintained better endothelial function and a transcriptomic profile enriched in metabolic regulation and extracellular matrix maintenance rather than senescence (1). This critical finding explains why non-specific senolytic strategies might be more effective in men, where plaque progression is driven by classical senescence pathways, whereas women might require therapies targeting metabolic resilience (1, 53).

### 5.2 Senomorphics: a strategy to modulate the SASP

Senomorphics are another class of compounds that do not kill senescent cells but instead modulate their phenotype, primarily by inhibiting the harmful SASP (54). This approach “disarms” the commander without eliminating it. A key example is rapamycin, an mTOR inhibitor, which has been shown to reduce the secretion of pro-inflammatory SASP components (55). This may be beneficial in situations where the cell cycle arrest of senescent cells is still desired (e.g., for tumor suppression) but their collateral inflammatory damage is not.

### 5.3 Targeting upstream drivers: from metabolic control to pathway-specific inhibitors

An alternative strategy is to prevent endothelial cell senescence at its source. This approach is particularly pertinent given the findings discussed in Section 4, where downstream blockage of the cell cycle (e.g., via CDK4/6 inhibition) paradoxically exacerbates the senescent phenotype. Therefore, therapeutic intervention must occur upstream of the cell cycle arrest. This involves targeting primary stressors and signaling pathways, such as lifestyle interventions and pharmacological control of metabolic risk factors to reduce the RBP4 load. It may also involve the development of specific inhibitors against pathways like TLR4 or JAK/STAT to block pro-senescent signals from molecules like RBP4 (56).

### 5.4 Precision clearance via antibody-drug conjugates: a novel frontier

While current senolytics act systemically, the field of oncology offers a precision medicine blueprint potentially applicable to atherosclerosis: Antibody-Drug Conjugates (ADCs). As reviewed by Wang et al., ADCs can deliver cytotoxic payloads specifically to cells expressing unique surface antigens, inducing immunogenic cell death (ICD) and transforming a “cold” immune environment into a “hot” one (2). Developing ADCs requires the identification of surface markers exclusively expressed on senescent cells, distinct from those on acutely activated or inflamed endothelium (e.g., VCAM-1). Targeting bona fide “seno-antigens” (such as B2M or novel membrane-bound proteomes) would allow for the precise ablation of the “commander” cells within the plaque, avoiding off-target toxicity to healthy but activated endothelial cells and mitigating the systemic side effects associated with broad-spectrum Bcl-2 inhibitors (2).

## 6 Conclusion: the senescent endothelial cell—a targetable central node in atherosclerosis

This report has systematically repositioned the senescent endothelial cell from a correlate of vascular aging to a central commander of atherosclerotic plaque development and instability. Through a complex and redundant communication arsenal including the SASP and extracellular vesicles, the sEC actively directs the pro-atherogenic behavior of immune cells and VSMCs, effectively engineering a pathological microenvironment.

This command function is driven by an interconnected network of signaling pathways. While the classic p53/p21 and p16/Rb pathways establish the senescent state, upstream regulators like mTOR and NF-κB fuel the inflammatory SASP. Crucially, the RBP4 signaling axis emerges as a paramount mechanism, providing a direct molecular link between systemic metabolic disease and local vascular senescence. Its dual TLR4 and STRA6 receptor pathways enable RBP4 to launch a convergent assault of inflammatory and metabolic signals, thereby cementing the pathological role of the sEC.

This nuanced understanding of the sEC as a “commander” opens new frontiers for therapy. Whether through clearance with senolytics, disarming with senomorphics, or prevention by targeting upstream drivers like RBP4, strategies aimed at “dethroning” the commander hold immense promise for the future treatment and prevention of atherosclerosis. Targeting this central, druggable node may prove more effective than addressing its numerous downstream consequences individually.
